# From injury events to rescue burden: accident patterns, health outcomes, and prevention in mountain outdoor physical activities—a scoping review

**DOI:** 10.3389/fpubh.2026.1930989

**Published:** 2026-09-10

**Authors:** Huan Tang, Fateh Zereg, Ruoqin Mo

**Affiliations:** 1 Chengdu Sport University, Chengdu, China; 2 Changchun Normal University, Changchun, China

**Keywords:** accidents, emergency evacuation, illness, injury, mountain outdoor sports, scoping review, search and rescue

## Abstract

**Background:**

Mountain outdoor physical activities generate accidents, traumatic injuries, acute illnesses, fatalities, navigation failures, environmental exposures, technical entrapments, and search-and-rescue or evacuation demands. This review mapped the international empirical evidence and examined how activity-specific incidents become rescue-system burdens.

**Methods:**

Following JBI guidance and PRISMA-ScR, we searched PubMed, Web of Science Core Collection, Scopus, and EBSCOhost for primary studies published between 1 January 2005 and 1 August 2026, without language restrictions. Eligible studies reported accidents, injuries, illnesses, fatalities, rescues, or evacuations in mountain, alpine, high-altitude, wilderness, snow, trail, or canyon settings. Data were charted on study characteristics, activities, exposures, outcomes, and rescue features. Methodological quality was assessed using appropriate JBI checklists or PROBAST, followed by descriptive and thematic synthesis.

**Results:**

Sixty-six studies were included. All 66 reported an explicit participant, casualty, mission, event, or case count, but most did not provide a comparable participation-exposure denominator. Walking and endurance activities were dominated by falls, exhaustion, navigation failure, and non-traumatic illness; vertical and technical activities by falls from height, protection or descent errors, and entrapment; high-speed and mechanically assisted activities by high-energy trauma; winter and avalanche activities by collision, burial, and time-critical rescue; and canyoning by combined trauma, drowning, hypothermia, and difficult evacuation. Uninjured rescue, inaccurate location, environmental amplification, and prolonged extraction recurred across activity groups.

**Conclusion:**

Mountain emergencies extend beyond injury to encompass illness, environmental exposure, navigation failure, technical entrapment, and uninjured rescue. They can be conceptualized as a descriptive rather than causal pathway from preparation and exposure to event onset, response, evacuation, and outcomes. Heterogeneous definitions and limited exposure denominators hinder cross-activity comparisons; priorities include standardized measures, linked rescue–clinical datasets, and prospective intervention evaluation.

## Highlights

- Evidence distribution: evidence was geographically concentrated, predominantly retrospective and registry-based, and rarely included comparable exposure denominators.
- Activity-specific patterns: incident mechanisms, health outcomes, and rescue demands differed substantially across activity groups.
- Cross-cutting factors: environmental, participant-related, and technological or organizational factors interacted across the accident-to-rescue pathway.
- Research priorities: standardized definitions and denominators, time-stamped rescue data, linked rescue–clinical registries, and prospective intervention evaluation are needed.

## 1 Introduction

Mountain outdoor physical activities have evolved substantially over recent decades, extending beyond traditional specialist and expeditionary practices to encompass diverse forms of recreation, endurance competition, and nature-based tourism. This development reflects not only increased participation but also the emergence, diversification, and progressive transformation of adventure physical activities in natural environments, including changes in their purposes, modes of practice, and relationships with technology and organized provision (1). Mountaineering and hiking, for example, now occupy overlapping recreational and tourism contexts (2, 3), while participation encompasses heterogeneous populations, including adolescents, women, middle-aged and older adults, and people with widely differing levels of experience and preparation (4, 5). At the same time, organized events, mountain-bike facilities, electrically assisted equipment, commercial guiding, and digital navigation have altered how mountain environments are accessed and negotiated. Consequently, the safety burden is also heterogeneous: frequently practiced activities such as hiking, alpine skiing, trail running, and mountain biking account for substantial numbers of incidents, whereas more technically demanding activities may involve severe trauma, difficult extraction, or time-critical rescue (6, 7).

Safety outcomes in mountain environments arise from interacting factors that can be considered across three broad domains. Environmental factors include terrain, altitude, weather, temperature, and darkness, all of which can alter exposure and constrain movement or rescue. Participant- or patient-related factors include injury and acute illness, but also fatigue, physical capacity, experience, preparedness, navigation, and the ability to self-evacuate. Technological and organizational factors encompass equipment, communication and positioning technologies, activity organization, access to assistance, search-and-rescue capability, evacuation, and transport to definitive care (6). These domains should not be understood as independent causes. Systemic accidentology has shown that mountain accidents and near misses may develop through dynamic sequences in which multiple direct and indirect contributing factors interact rather than through a single precipitating event (8). Thus, an apparently minor injury, exhaustion, loss of route, equipment problem, or deterioration in weather may combine with limited mobility, communication difficulty, or delayed access to assistance to transform a manageable situation into a prolonged search, rescue, and medical-evacuation event.

The evidence base reflects this multidimensional accident pathway but is distributed across surveillance systems that capture different components of it. Emergency-department and trauma-registry studies provide detailed information on participant- or patient-related outcomes such as diagnoses, anatomical injury patterns, and clinical severity, but often provide less information on environmental circumstances or the sequence leading to rescue (7). Search-and-rescue and police records can more directly characterize environmental and technological or organizational dimensions, including location, access, exposure, navigation problems, evacuation requirements, and operational resource use; importantly, they also capture people who may be lost, stranded, exhausted, cold, or otherwise unable to continue despite having no major traumatic injury (9, 10). Forensic and mortality records emphasize the most severe outcomes, whereas event medical systems, ski-patrol records, national injury-surveillance systems, and participant surveys may better represent other portions of the severity spectrum. These complementary perspectives broaden understanding of mountain safety, but differences in case definitions, denominators, activity classifications, and recorded variables continue to limit direct comparisons across studies and activities (6–10).

A scoping review is therefore appropriate not because previous research has considered injury alone, but because evidence concerning environmental, participant/patient-related, and technological/organizational factors remains distributed across different activities, disciplines, data systems, and outcome definitions. Integrating these perspectives can clarify which features are activity-specific, which mechanisms recur across settings, and where surveillance or prevention remains incomplete. Accordingly, this review addressed four questions: (1) how is the evidence distributed across regions, activities, designs, data sources, populations, and outcomes; (2) how do accident mechanisms, health outcomes, and rescue characteristics differ by activity; (3) how do environmental, participant- or patient-related, and technological or organizational factors recur and interact across accident and rescue sequences; and (4) which priorities for prevention, surveillance, and emergency-service design follow from the international evidence?

## 2 Methods

### 2.1 Design and reporting framework

This scoping review followed PRISMA-ScR, the framework of Arksey and O’Malley, and updated Joanna Briggs Institute methodological guidance (11–13). Because the included studies differed substantially in activity, setting, event definition, data source, denominator, and outcome, the review was designed for evidence mapping, activity-stratified synthesis, and thematic integration rather than pooled effect estimation. The protocol was not prospectively registered.

### 2.2 Eligibility framework

Eligibility was defined using the Population–Concept–Context framework. The population comprised people participating in mountain outdoor physical activities, accident casualties, injured or ill patients, fatalities, and persons requiring rescue. The concept included accidents, injuries, illnesses, fatalities, near-miss or stranded events, search and rescue, evacuation, survival, risk factors, and prevention. The context comprised mountain, alpine, high-altitude, wilderness, snow, canyon, trail, mountain-activity park, outdoor-event, and other remote natural environments.

Primary empirical studies were eligible, including retrospective or prospective observational studies, registry analyses, cross-sectional studies, case–control studies, event medical records, rescue archives, mortality investigations, prediction-model studies, and participant surveys. Activities included hiking, mountaineering, rock climbing, via ferrata, alpine and backcountry skiing, snowboarding, ski touring, mountain biking, electric mountain biking, trail running, canyoning, snowmobiling, and avalanche-exposed recreation. Reviews, commentaries, editorials, conference abstracts without full data, occupational or military mountain work, indoor-only activity, ordinary road traffic incidents, and studies limited to physiology or performance without a safety outcome were excluded. Mixed indoor/outdoor climbing studies were retained when outdoor climbing was represented and the data informed the review question; in broad fatality datasets, intentional self-harm was excluded from the mountain-activity safety synthesis. Eligibility was determined after bibliographic deduplication; duplicate records were removed before title and abstract screening and were not counted as eligible studies.

### 2.3 Information sources and search strategy

PubMed, Web of Science Core Collection, Scopus, and EBSCOhost were searched for studies published from 1 January 2005 to 1 August 2026. No language filter was applied in the updated search. Non-English full texts were assessed with translation support and cross-checking of the original methods, tables, and numerical results. Search concepts combined environment terms (mountain, alpine, wilderness, backcountry, high altitude, trail, canyon), activity terms (hiking, mountaineering, climbing, skiing, snowboarding, mountain biking, trail running, canyoning, snowmobiling, avalanche), and outcome terms (accident, injury, trauma, illness, fatality, mortality, rescue, evacuation, survival, prevention). Grey literature was not excluded solely because of publication format when a database-retrieved report contained sufficient empirical methods and results; however, no separate search of organizational or government websites was undertaken. Only records identified through the four bibliographic databases contributed to the final selection. Complete database-specific strategies and retrieved record counts are reported in Supplementary Table S2.

### 2.4 Study selection

Records were deduplicated and screened independently by two reviewers in two stages: title/abstract screening and full-text assessment. Before formal screening, the reviewers calibrated the eligibility criteria on a random sample. Cohen’s kappa was calculated for a 20% random sample; agreement was good at title/abstract screening (*κ* = 0.75) and full-text assessment (*κ* = 0.78) (14). Disagreements were resolved by discussion and, when necessary, adjudication by a third reviewer.

### 2.5 Data charting and evidence-independence assessment

A standardized charting form captured bibliographic details, study setting, activity, design, data source, study period, participant or case count, outcome domains, exposure denominator, accident mechanisms, health outcomes, rescue characteristics, risk modifiers, and prevention implications. For this review, an uninjured rescue was coded only when the source classified a person or mission as having no documented traumatic injury or acute illness at the time of rescue; the term did not imply absence of hazard, need for assessment, assistance, or evacuation. Each of the 66 included reports was assigned a unique study identifier. Reports drawing on the same registry, geography, study period, activity, or participant population were flagged as potentially overlapping and were interpreted together; raw case totals from potentially overlapping reports were not treated as independent counts. Study characteristics, exposure bases, principal findings, and appraisal profiles are presented in Supplementary Table S1.

### 2.6 Critical appraisal

All 66 included studies underwent design-appropriate critical appraisal. JBI tools were applied to 65 studies: 28 analytical cross-sectional, 22 prevalence or occurrence, eight case-series, six cohort, and one case–control study. PROBAST was applied to the remaining prediction-model study (15, 16). Item-level responses were recorded as yes, no, unclear, or not applicable, as permitted by the relevant instrument. Because the instruments differ in structure and decision framework, no cross-tool numerical score, percentage threshold, or *post hoc* high/moderate/low quality grade was created. Appraisal findings were used to qualify interpretation and did not determine study eligibility. The study-level instrument and item profile are reported in Supplementary Table S1.

### 2.7 Synthesis

Evidence was mapped by publication year, region, design, data source, activity domain, sample or case-count reporting, and exposure-denominator class. Activity domains were walking/endurance, vertical/technical, high-speed/mechanically assisted, winter/avalanche, canyoning/special environment, and multisport/rescue-system research. All studies were required to report a study population, event count, casualty count, mission count, or other explicit sample indicator. Exposure denominators were coded separately according to whether a participation-based denominator was clear or derivable, a weighted case estimate without participation exposure was reported, or no participation-exposure denominator was available. Cross-cutting themes were developed iteratively across the accident, exposure, rescue, and evacuation pathway. Critical-appraisal findings were considered alongside study design and data-source limitations when interpreting the evidence. Descriptive associations were not interpreted as causal effects.

## 3 Results

### 3.1 Study selection and overall evidence profile

**Figure 1:**
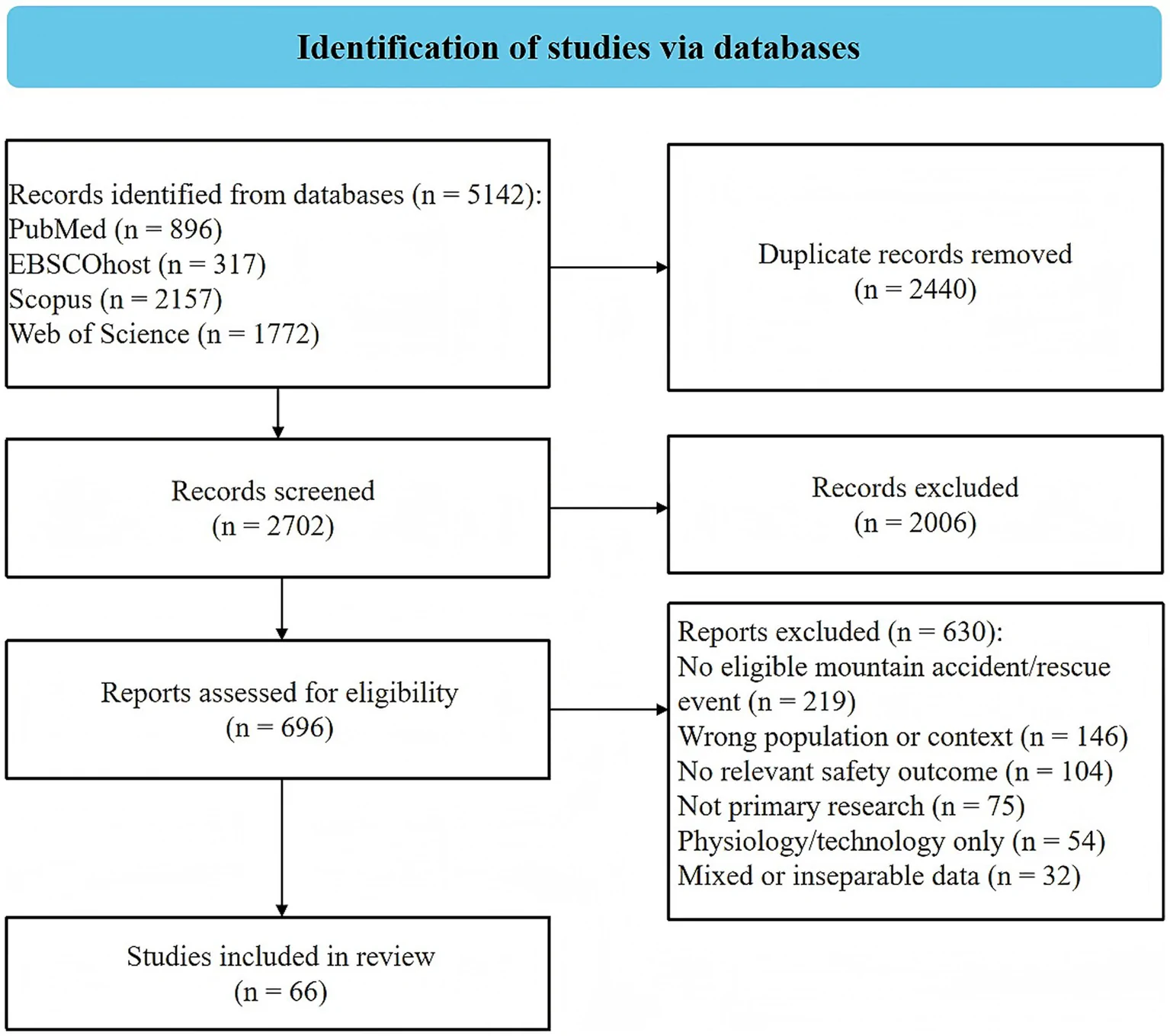
Study selection flow diagram. Four bibliographic databases identified 5,142 records. After 2,440 duplicates were removed, 2,702 records were screened, 696 full-text reports were assessed, 630 were excluded by the stated categories, and 66 studies were included. No records were added through other identification methods.

The database searches identified 5,142 records: 896 from PubMed, 317 from EBSCOhost, 2,157 from Scopus, and 1,772 from Web of Science Core Collection. After 2,440 duplicates were removed, 2,702 records underwent title and abstract screening, of which 2,006 were excluded. Six hundred ninety-six full-text reports were assessed for eligibility; 630 were excluded because there was no eligible mountain accident or rescue event (*n* = 219), the population or context was ineligible (*n* = 146), no relevant safety outcome was reported (*n* = 104), the report was not primary research (*n* = 75), it addressed physiology or technology without a safety outcome (*n* = 54), or outdoor results could not be separated (*n* = 32). Sixty-six studies were included (Figure 1). Study characteristics, population and exposure bases, and appraisal profiles are provided in Supplementary Table S1, and database-specific search strategies are provided in Supplementary Table S2 (9, 17–81).

Studies were published from 2005 to 2026; 44 of 66 were published from 2020 onward and 25 from 2024 to 2026. Forty-three studies were conducted in Europe, 15 in North America, five in Asia, two in South America, and one in Africa. The activity distribution comprised 15 vertical/technical studies, 15 multisport or rescue-system studies, 15 winter/avalanche studies, 11 walking/endurance studies, eight high-speed/mechanically assisted studies, and two canyoning studies. Design adjudication identified 28 analytical cross-sectional studies, 22 prevalence or occurrence studies, eight case-series studies, six cohort studies, one case–control study, and one prediction-model study. The activity-specific synthesis is summarized in Table 1.

**Table 1:** Activity-specific mechanisms, health outcomes, rescue-system signatures, and evidence limitations.

Activity domain and included activities (studies)	Principal mechanisms	Health outcomes	Rescue-system signature	Evidence limitations
Walking/endurance: hiking, trekking, trail running, endurance events (*n* = 11)	Falls, descent errors, exhaustion, navigation failure, weather change, training-load imbalance	Lower-limb injury; dehydration, gastrointestinal, cold, altitude, or cardiac illness	Large mission volume; lost, exhausted, overdue, and uninjured casualties	Few exposure-based cohorts; rescue studies predominantly reported mission or case counts; demographic bands varied.
Vertical/technical: rock climbing, via ferrata, mountaineering, glacier travel (*n* = 15)	Falls from height; protection, belay, descent, or rope-team failure; rockfall; entrapment; exhaustion	Head, spinal, pelvic, lower-limb, and multisystem trauma; altitude illness	Rope rescue, helicopter access, prolonged extraction, and uninjured entrapment	Registry and case-series evidence predominated; overlapping national registries and scarce participation denominators limited comparative risk estimates.
High-speed/mechanically assisted: MTB, downhill MTB, e-MTB (*n* = 8)	Loss of control; failed jump or landing; over-the-handlebars; collision	Upper-extremity, shoulder, clavicle, head, torso, and spinal injury; fracture, admission, surgery, or disability	High transport and trauma-care demand; occasional catastrophic disability	Emergency and trauma systems captured treated cases; participation exposure was usually unavailable, including for electric-bike comparisons.
Winter/avalanche: skiing, snowboarding, ski touring, freeriding, avalanche-exposed travel (*n* = 15)	High-speed fall or collision; jump/landing failure; avalanche burial	Knee, wrist, shoulder, and head injury; suffocation, trauma, hypothermia	Minute-sensitive companion rescue, beacon localization, organized rescue, and aviation constraints	Some resort studies reported skier-days, whereas avalanche databases selected severe or buried cases; severity and process definitions varied.
Canyoning/special environment: canyoning and canyon rescue (*n* = 2)	Slippery falls; jumps; rope error; water entrapment; drowning	Lower-limb and multisystem trauma; hypothermia; drowning	Communication difficulty, helicopter dependence, and prolonged technical extraction	Only two studies were available; both relied on case or mission counts without a comparable participation denominator.
Multisport/rescue system: National parks, regional rescue systems, multisport registries (*n* = 15)	Falls, illness, getting lost, entrapment, exhaustion, weather, technical inability	Mixed trauma and illness; substantial uninjured-rescue burden	Ground, stretcher, rope, and helicopter evacuation; high personnel and time demand	Activities, definitions, and registry coverage were heterogeneous; overlapping sources and absent participation exposure precluded pooled incidence.

Study populations ranged from event participants and uninjured controls to rescue casualties, emergency-department patients, hospitalized patients, and fatalities; an aggregate population total was not calculated because units of analysis differed and several reports drew on overlapping registries. Age-specific reporting used non-equivalent cut-points. Separate younger-age data included 998 minors (10%) in the Polish rescue cohort (79), 339 patients aged <18 years (24.7%) in a US climbing study (53), 68 patients aged <15 years (9.3%) in a Chinese ski-resort study (52), and 60 patients aged <15 years (7%) in a Swiss helicopter-rescue cohort (69). In the Norwegian case–control study, participants aged 13–20 years constituted 42.5% of injury cases and 25.6% of controls (72). Sex/gender composition also varied: women constituted 51.6% of a national US trail-injury sample (62), whereas men constituted 82.0% of crevasse casualties (18), 82.6% of French mountaineering casualties (67), and 93.1% of patients with mountain-bike-related spinal cord injury (60). Heterogeneous age bands and study populations precluded pooled demographic estimates.

### 3.2 Data-source architecture and what it reveals

Search-and-rescue or police registries were the most common source (25 studies), followed by event or venue medical records (six), national injury-surveillance systems (five), hospital/emergency/trauma registries (five), and avalanche-specific databases (four). Additional studies used forensic or mortality records, national-park rescue data, participant surveys, public reports, or linked multisource datasets. Rescue registries captured lost, stranded, exhausted, and uninjured casualties, response mode, and extraction. Clinical systems provided more precise diagnoses, operations, admissions, and anatomical injury patterns. Avalanche databases captured burial depth, airway status, companion rescue, beacon use, rescue interval, and survival. These systems described complementary segments of the accident-to-rescue pathway rather than interchangeable populations.

Comparability was maintained by retaining each study’s original unit of analysis and denominator, separating survey-weighted case estimates from participation exposure, mapping reported outcomes to common descriptive domains, and restricting comparisons to studies with like units or data sources. Seven potentially overlapping registry clusters involving 26 reports were identified; their case totals were not summed as independent observations. Clinical and national surveillance sources therefore informed injury type and healthcare disposition (20, 30, 53, 58–60, 62, 71), rescue sources informed mission characteristics and non-injury presentations (9, 21, 23, 25, 31, 36, 37, 44, 49, 50, 67, 74, 76), and fatality or avalanche sources informed severe mechanisms and time-dependent survival (22, 29, 33–35, 38, 40, 41, 45–47, 56, 57, 61, 64). No pooled incidence estimate was calculated across these systems.

### 3.3 Activity-specific accident patterns

#### 3.3.1 Walking and endurance activities

Walking and endurance studies included hiking, high-altitude trekking, trail running, and mountain ultramarathons. Falls, particularly during descent, were the predominant traumatic mechanism, and ankle, knee, foot, and lower-leg injuries recurred across settings (17, 22, 24, 51, 55, 63). In 4,831 mountain-race participants followed over 19,684 running hours, 28 injuries occurred (1.6 per 1,000 h); 75% were minor and 78% involved the lower limb (17). In a 285-runner ultramarathon cohort, 89 runners had 131 medical encounters, comprising 82 illnesses and 49 injuries; illness and injury prevalence were 22.5 and 14.7%, respectively (39). Rescue datasets additionally recorded lost, overdue, exhausted, or otherwise unable-to-self-evacuate hikers without major injury (25, 36, 50, 68, 74).

#### 3.3.2 Vertical and technical activities

Rock climbing, via ferrata, glacier travel, and high-altitude mountaineering combined height exposure, protection-system dependence, and difficult evacuation. Among 321 complete crevasse-accident cases, 55% were classified as NACA 0–1, 9.4% as NACA ≥4, and 21 people died (6.5%) (18). In 7,819 people evacuated during French mountaineering incidents, 4,178 (53.5%) were uninjured, 2,537 (32.4%) were injured, 696 (8.9%) were ill, and 377 (4.8%) died (67). Across the remaining studies, recurrent mechanisms included falls from height, belay or descent errors, rope-team failure, rockfall, entrapment, and being technically blocked (23, 30, 31, 44, 53, 54, 59). High-altitude fatalities included trauma, altitude illness, hypothermia, cardiac events, and exhaustion, with distributions varying by route, ascent or descent phase, altitude, age, and rescue accessibility (24, 29, 35, 40, 41, 46, 57).

#### 3.3.3 High-speed and mechanically assisted activities

Mountain biking, downhill mountain biking, and electric mountain biking were characterized by loss of control, failed jumps or landings, projection over the handlebars, and collision. A national US surveillance study estimated 35,260 mountain-bike fractures; 32.8% involved the upper extremity, 26.7% the shoulder, and 23.7% the trunk, while 21.7% were hospitalized and 19.5% were classified as polytrauma (20). In a Canadian series of 58 mountain-bike-related spinal cord injuries, 27 were motor-complete, 31 motor-incomplete, 67.2% involved levels C3–C7, and 82.8% required surgery (60). Other emergency and bike-park studies likewise reported prominent upper-extremity, shoulder-girdle, clavicle, head, torso, and spinal injuries (28, 43, 58, 71). Injured electric-mountain-bike riders were older and had a different anatomical distribution from conventional riders, but the available case series did not provide exposure-adjusted comparative risk (65).

#### 3.3.4 Winter and avalanche activities

Alpine skiing and snowboarding studies showed different anatomical profiles: knee and lower-leg injuries were more frequent among skiers, whereas wrist, forearm, shoulder, and other upper-extremity injuries were more frequent among snowboarders (19, 32, 42, 52, 72, 75). The largest exposure-based ski study recorded 43,283 patients over 98.1 million skier-days, corresponding to 0.44 injuries per 1,000 skier-days (75). Among 1,643 critically buried avalanche victims in Switzerland, 878 survived and 765 died (46.6%); between the earliest decade and the full 1981–2020 period, median rescue time decreased from 45 to 25 min and survival increased from 43.5 to 53.4% (64). The same study found that the steep decline in survival began within approximately the first 10 min of burial. Other avalanche studies also associated survival with burial depth, airway status, time of day, companion rescue, beacon use, and professional-rescue arrival (33, 34, 45, 61).

#### 3.3.5 Canyoning and water–terrain interaction

Canyoning combined slippery-terrain falls, jumps, rope manoeuvres, water entrapment, drowning, hypothermia, and prolonged technical extraction. In the Italian national rescue study, 344 operations involved 569 people: 263 (46.2%) were uninjured, 253 (44.5%) had trauma, 46 (8.1%) had medical or environmental conditions, and 39 (6.9%) died; median rescue duration was 160 min (interquartile range 100–270) (76). Lower-extremity trauma predominated, whereas fatal outcomes were mainly associated with trauma and drowning (48, 76).

### 3.4 Cross-cutting themes

#### 3.4.1 Participation volume and event severity create a dual burden

High-participation activities generated the largest absolute incident and mission counts, whereas smaller technical, high-speed, avalanche, and canyon cohorts contained larger proportions of complex rescue or severe outcomes. For example, hiking accounted for 63.0% of 3,257 rescue operations in Catalonia, but the same registry included 696 uninjured casualties (21.4%), 164 life-risk cases (5.0%), and 115 deaths (3.5%) across activities (21). National injury-surveillance estimates reached 1,146,630 musculoskeletal trail-sport injuries (62), while smaller condition-specific cohorts documented outcomes such as spinal cord injury (60) or critical avalanche burial (64). Absolute burden and event severity were therefore reported on different statistical bases and were not combined into a single activity ranking.

#### 3.4.2 A descriptive accident-to-rescue pathway

**Figure 2:**
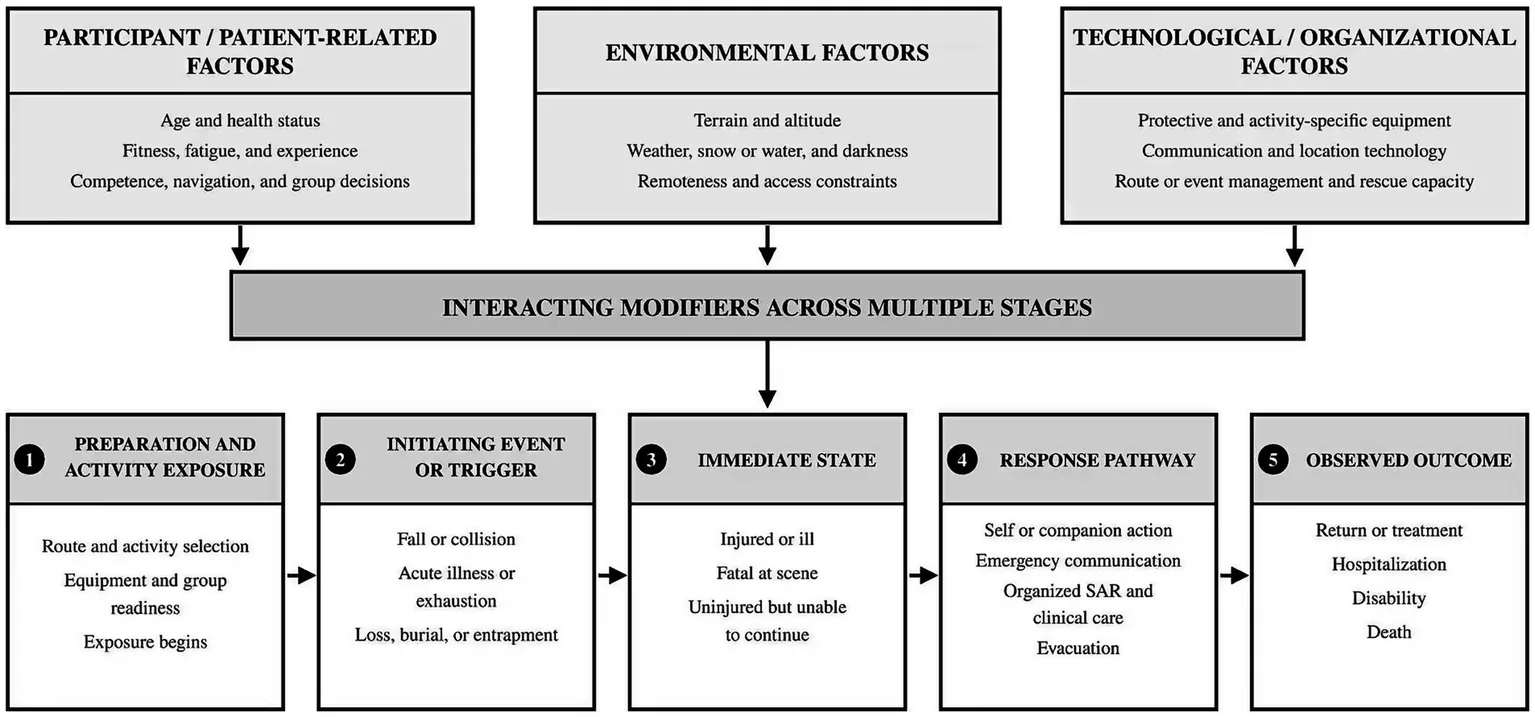
Descriptive accident-to-rescue pathway used to organize the evidence synthesis. Environmental, participant- or patient-related, and technological or organizational factors may influence multiple stages. Arrows show temporal ordering in the review synthesis, not validated causal effects.

Across activities, recurrent event sequences extended from activity exposure and an initiating event through an immediate state (injury, illness, death, loss, burial, entrapment, or inability to continue), companion action and emergency communication, organized search and rescue, clinical care, evacuation, and final disposition. Environmental conditions could modify several stages, as could participant- or patient-related factors and technological or organizational factors (Figure 2). Figure 2 is a descriptive framework developed from the mapped evidence and conceptually informed by the systemic accident-sequence perspective of Vanpoulle et al. (10); its arrows indicate temporal ordering rather than validated causal effects.

#### 3.4.3 Uninjured rescue is a measurable system outcome

Using the source-based operational definition above, uninjured rescue denoted no documented traumatic injury or acute illness at the time of rescue; it did not mean that no hazard, clinical assessment, assistance, or evacuation was present. Such casualties constituted a substantial recorded outcome in several rescue systems. The US National Park Service dataset classified 51,541 of 78,488 people (65.7%) as neither ill nor injured (36); corresponding proportions were 4,178 of 7,819 (53.5%) in French mountaineering rescues (67), 263 of 569 (46.2%) in Italian canyoning rescues (76), and 696 of 3,257 (21.4%) in Catalonian mountain rescues (21). Reported circumstances included being lost, overdue, technically blocked, exhausted, overtaken by darkness or weather, or unable to continue (23, 25, 31, 37, 44, 49, 50, 54, 74).

#### 3.4.4 Equipment effects were context-dependent

Equipment-related findings were conditional on use context. Among 278 totally buried avalanche victims, median burial time was 20 min with a transceiver and 102.5 min without one; mortality was 53.8 and 68.0%, respectively (45). Helmet adoption coincided with fewer head injuries in one ski setting (32), whereas missing or incorrectly used protection systems were documented in climbing and via ferrata fatalities (23, 54). Electric assistance was associated with an older injured rider profile, but the available data did not include comparable exposure denominators (65).

#### 3.4.5 Time, location, and on-scene capability shape outcome

Time-to-rescue measures were most consistently reported in avalanche and specialist aviation studies. In the Swiss avalanche series, median rescue time decreased from 45 to 25 min over four decades (64). In 842 Swiss helicopter external-cargo rescue cases, median take-off and on-scene intervals were 5 and 26 min, respectively; among patients with NACA ≥4, 33% reached hospital within 60 min, 35% within 61–90 min, and 32% after >90 min (69). Other glacier, canyon, climbing, and high-altitude studies recorded delays related to darkness, weather, imprecise location, vertical terrain, water, remote access, or restricted aviation windows (18, 33, 34, 37, 61, 76).

### 3.5 Measurement and reporting heterogeneity

All 66 studies reported an explicit participant, casualty, patient, mission, accident, fatality, or event count. Seventeen studies reported some participation-based measure, including participants, person-time, visits or rides, permits, skier-days, event exposure, or a comparable basis; completeness and stratification nevertheless varied. Five national surveillance studies reported survey-weighted estimates of treated cases without measuring the population exposed. The remaining 44 studies were based on observed cases, missions, injuries, or fatalities only. Thus, 49 of 66 studies lacked a participation-exposure denominator, and their case proportions describe case mix or system burden rather than activity incidence. Study-level exposure bases are reported in Supplementary Table S1.

Injury-severity measurement was also heterogeneous. Nine studies used NACA or a simplified NACA-based scheme, three directly used UIAA-based classifications, one additional study approximately mapped a local four-level outcome to UIAA categories, and one used the Injury Severity Score; most others used local legal or administrative categories or pragmatic outcomes such as admission, surgery, evacuation, or death. The publicly available UIAA Medical Commission framework specifies common reporting for injury location, severity, and fatality risk (82), but direct use of UIAA-based classifications was identified in only three included studies. Across the evidence base, units alternated among people, accidents, missions, injuries, fatalities, visits, and weighted emergency-department estimates, and time-stamped process variables were inconsistently reported outside avalanche and specialist aviation datasets.

### 3.6 Critical appraisal

All 66 included studies underwent design-appropriate critical appraisal. JBI checklists were applied to 65 studies: 28 analytical cross-sectional, 22 prevalence or occurrence, eight case-series, six cohort, and one case–control study; PROBAST was applied to the single prediction-model study. The tool-specific JBI overall judgments were Include for 60 studies and Seek further info for five. The prediction-model study was judged to have high risk of bias and high concern for applicability. These judgments were not converted to numerical scores or compared across instruments, and no study was retrospectively excluded on the basis of appraisal (15, 16).

## 4 Discussion

### 4.1 Principal findings

This review identifies activity-specific but not exposure-comparable patterns across 66 studies. Walking and endurance findings repeatedly involved falls during descent, lower-limb injury, fatigue, and non-traumatic illness (17, 39, 51, 55, 63); vertical and technical activities emphasized falls from height, protection or descent failures, entrapment, and difficult evacuation (18, 23, 30, 54, 67); high-speed activities involved fractures, head or spinal injury, and high-energy mechanisms (20, 28, 60, 71); and winter, avalanche, and canyoning studies highlighted collision, critical burial, water exposure, and prolonged extraction (32, 45, 64, 75, 76). Agreement across rescue, clinical, and surveillance sources strengthens these descriptive profiles, but differences in case ascertainment and denominators preclude ranking activities by risk.

The principal interpretive distinction is between system burden and population risk. Rescue registries quantify casualties and resource demand (36, 67, 79), whereas national injury systems estimate treated cases (20, 62); only studies with participation- or time-based denominators can estimate incidence (17, 75, 77). In the updated evidence base, 17 studies reported some participation-based measure, five provided survey-weighted case estimates without activity exposure, and 44 described observed cases, missions, injuries, or fatalities only. Thus, comparisons of case mix are supported, whereas cross-activity incidence comparisons generally are not.

### 4.2 Conceptual contribution: an injury–exposure–rescue pathway

The injury–exposure–rescue pathway in Figure 2 is an interpretive synthesis, not a validated causal model. It is consistent with Vanpoulle et al.’s (10) systemic account, but the included studies support only individual links: burial time and transceiver use with avalanche survival (45, 64); terrain and access with rescue complexity (18, 69, 76); and becoming lost, exhaustion, or technical blockage with rescue activation (36, 67, 79). These findings justify organizing the evidence temporally; they do not establish that modifying any single component will necessarily improve outcomes.

Uninjured rescue should therefore be treated as a surveillance outcome rather than a distinct causal mechanism. Within Catalonian, French mountaineering, and Italian canyoning registries, the same broad circumstances—including falls, technical blockage, becoming lost, exhaustion, or environmental exposure—were associated with outcomes ranging from uninjured rescue to injury or death (21, 67, 76). Whether a person remains uninjured can depend on mobility, shelter, health, location accuracy, companion action, weather, and rescue delay (64, 69, 73). Recording uninjured cases separately is useful for describing rescue workload, but it does not show that these missions are mechanistically separate or necessarily preventable.

### 4.3 Prevention and emergency-service implications

The included studies directly observed recurrent associations involving descent falls, navigation or becoming lost, protection systems, equipment use, and avalanche burial (23, 45, 51, 54, 61, 63, 67, 72, 79). Most were retrospective and did not evaluate preventive interventions. Route matching, turnaround thresholds, weather interpretation, partner-rescue training, and equipment checks should therefore be presented as practice implications inferred from the observed patterns, not as interventions proven by this review. The clearest time-dependent evidence concerned avalanche burial, for which companion rescue, transceiver use, and shorter burial intervals were associated with survival (45, 64).

Evidence on rescue modality was more limited. Included mountain studies documented prolonged access or evacuation and the need for on-scene assessment, analgesia, or stabilization, but did not establish a causal advantage of air over ground rescue (18, 25, 37, 69, 76). In an external Korean cohort—not a mountain-activity study—Lee and Lee reported survival to discharge of 85.3% among 34 severely injured patients transported by physician-staffed helicopter and 62.8% among 105 transported by ground ambulance (83). The retrospective design, small sample, ≥30-km selection criterion, and different trauma-system context restrict causal inference and transferability. This evidence supports studying transport time, prehospital capability, and modality together; it does not justify a general claim that helicopter rescue is superior.

Exposure-based surveillance is most feasible where starts, visits, skier-days, or activity-hours are routinely recorded, as in races, ski areas, and prospective participation cohorts (17, 39, 75, 77). By contrast, proposals for location interoperability, standardized time stamps and triage, air–ground coordination, and linked rescue–clinical records are author-derived system priorities prompted by reporting gaps. They should be evaluated against patient outcomes, response intervals, resource use, and unintended effects before being treated as established best practice.

### 4.4 Research agenda

Two empirical gaps are especially clear. First, most studies lacked participation exposure; second, injury severity and rescue-process variables were inconsistently classified. Nine studies used the National Advisory Committee for Aeronautics (NACA) score or a simplified variant, while direct use of UIAA-based classification was uncommon despite the availability of the UIAA framework (82). On this basis, we propose—not infer as an established consensus—a minimum dataset separating case numerators from exposure denominators and recording activity, terrain, weather, competence, equipment, location, rescue intervals, clinical severity, and disposition. Linkage between rescue and clinical systems is a research priority because each source captures only part of the event sequence (25, 36, 67, 69).

The evidence base contained prospective surveillance, case–control analyses, and observational equipment studies (45, 72, 77), but few evaluated organizational or behavioral interventions. Consequently, digital location systems, route grading, public education, equipment training, trail design, and changes in rescue organization should be tested as hypotheses rather than recommended as proven solutions. Evaluations should prespecify exposure, process, and health outcomes and report implementation fidelity, selection effects, and possible unintended changes in participation or risk behavior.

### 4.5 Strengths and limitations

Strengths include broad activity coverage, integration of clinical and rescue data, explicit separation of case counts from exposure denominators, design-appropriate appraisal, and checks for overlapping registry cohorts. These choices accord with scoping-review guidance emphasizing transparent mapping of heterogeneous evidence rather than forced quantitative pooling (11–13). Appraisal used design-specific JBI instruments and PROBAST without converting them into a common numerical quality scale (15, 16).

Limitations include the database-only search, geographic concentration, predominance of retrospective records, inconsistent definitions, and incomplete exposure data. Although no language restriction was applied and eligible non-English full texts were included, the search concepts were primarily English and translation may have introduced interpretive uncertainty. National and organizational statistics, including recurring Swiss Alpine Club and Spanish Ministry of the Interior reports, can provide important surveillance information (84, 85); because government and organizational websites were not systematically searched, non-indexed official and grey literature may have been missed. Several included studies relied on public reports, administrative coding, or reconstructed fatality series (26, 46, 80), and overlapping registries meant that report counts could not always be treated as independent evidence. Meta-analysis was therefore not appropriate. Minor or self-managed events and near misses were probably under-ascertained, publication bias cannot be excluded, and the proposed pathway remains an interpretive framework requiring prospective testing (10). External emergency-care evidence, including Lee and Lee (83), was used only to contextualize questions about transport and care and was not treated as direct mountain-rescue evidence.

## 5 Conclusion

This scoping review shows that mountain emergencies should be understood not as isolated injury events, but as interconnected processes involving activity exposure, initiating events, immediate states, rescue responses, and eventual outcomes. These stages, together with the environmental, participant-related, and organizational factors that may influence them, are summarized in the descriptive accident-to-rescue pathway (Figure 2). This pathway is intended as an interpretive framework rather than a validated causal model. Although incident patterns vary across activities, non-traumatic illness, navigation failure, environmental exposure, technical entrapment, and difficult evacuation consistently contribute to rescue burden. Current evidence is limited by heterogeneous definitions, scarce exposure denominators, and fragmented rescue and clinical data, precluding robust comparisons of risk across activities. Future research should therefore prioritize standardized, context-sensitive reporting, linked longitudinal data, and prospective evaluation of preventive and response strategies. Ultimately, improving mountain safety requires a shift from injury-focused surveillance toward an integrated, pathway-based approach that connects prevention, preparedness, rescue, clinical care, and outcomes.

## Data Availability

The original contributions presented in the study are included in the article/Supplementary material, further inquiries can be directed to the corresponding author.
