# Supplementary material for: From injury events to rescue burden: accident patterns, health outcomes, and prevention in mountain outdoor physical activities—a scoping review
Source: Front Public Health. 2026 Sep 10;14:1930989. doi: 10.3389/fpubh.2026.1930989 (PMC13600856; doi:10.3389/fpubh.2026.1930989)
Supplement: Supplementary file 1 [file Table_1.docx]

**Supplementary Materials**

From Injury Events to Rescue Burden: Accident Patterns, Health Outcomes, and Prevention in Mountain Outdoor Physical Activities—A Scoping Review

Study identifiers S01–S66 are used consistently in this supplement. Each study was appraised with one design-appropriate instrument. Compact item-response profiles and tool-specific decisions are shown; the complete evidence audit is retained by the authors.

**Supplementary Table S1. Characteristics and design-matched critical appraisal of the 66 included studies**

| **Study** | **Country/region** | **Activity** | **Design, data source, and period** | **Population/sample or case count** | **Exposure basis** | **Appraisal instrument; item profile; tool-specific decision** |
| --- | --- | --- | --- | --- | --- | --- |
| S01. González-Lázaro et al., 2021 | Spain (Castilla y León) | 20–42 km mountain running races | Prevalence/occurrence; Questionnaire/self-report; 2015–2019. | 36 races; 6,167 entries and 4,831 analyzed runners; 28 injuries; 19,684 running hours. | Runner-hours and participants | JBI-Prev; Y5/N1/U3/NA0; Include |
| S02. Klocker et al., 2022 | Switzerland | Crevasse rescue in mountaineering/backcountry snow sports | Analytical cross-sectional; Swiss Alpine Club mountain-accident registry; 2010–2020. | 405 registered; 321 complete cases analyzed; fall depth available for 106. | Case/mission count only | JBI-ACS; Y4/N2/U2/NA0; Include |
| S03. Wallner et al., 2022 | Austria | Skiing and snowboarding collisions | Analytical cross-sectional; Police incident records; 2005–2018. | 30,503 collisions; 61,006 persons (52,430 skiers; 8,576 snowboarders). | Case/mission count only | JBI-ACS; Y4/N0/U4/NA0; Include |
| S04. Koehne et al., 2024 | United States | Mountain-bike riding | Prevalence/occurrence; NEISS emergency-department surveillance; 2013–2022. | 2,543 MTB injuries after exclusions; 853 sampled fractures (weighted n=35,260). | Weighted case estimate; no activity exposure | JBI-Prev; Y6/N0/U2/NA1; Include |
| S05. Martínez et al., 2025 | Catalonia, Spain | Hiking, climbing, MTB, snow activity, canyoning and other practices | Analytical cross-sectional; Rescue/incident records; 2011–2021. | 3,257 operations; 2,285 complete cases; final model n=2,091. | Case/mission count only | JBI-ACS; Y3/N2/U3/NA0; Include |
| S06. Zürcher et al., 2020 | Canton of Bern, Switzerland | Mountain hiking | Analytical cross-sectional; SAC, forensic, and prosecutor records; 2003–2018. | 124 hiking deaths identified; 77 fatal accidents analyzed (37 <1,800 m; 40 >1,800 m). | Case/mission count only | JBI-ACS; Y3/N2/U3/NA0; Include |
| S07. Lack et al., 2012 | Boulder County, Colorado, USA | Roped/unroped climbing, bouldering and mountaineering | Prevalence/occurrence; Rescue/incident records; 1998–2011. | 1,857 SAR incidents/2,198 victims; climbing subset: 345 incidents/428 victims. | Case/mission count only | JBI-Prev; Y6/N0/U3/NA0; Include |
| S08. Jones et al., 2014 | Fujisan, Yamanashi, Japan | Summer ascent/descent of Fujisan | Analytical cross-sectional; Police incident records; 1989–2008. | 155 incidents. | Case/mission count only | JBI-ACS; Y2/N3/U3/NA0; Include |
| S09. McDonough et al., 2020 | King County, Washington, USA | Mostly hiking; also climbing, snow sports and others | Prevalence/occurrence; Rescue/incident records; 2004–2017. | 1,425 county missions; SMR involved in 851; 552 included, involving 756 subjects. | Case/mission count only | JBI-Prev; Y4/N3/U2/NA0; Include |
| S10. Roi, 2021 | Andorra, Austria, France, Italy, Spain, Switzerland, UK | Fell, skyrunning and ultratrail running | Case series; Media/website case compilation; 2008–2019. | 51 fatalities. | Case/mission count only | JBI-CS; Y5/N2/U3/NA0; Seek further info |
| S11. Goodrum et al., 2025 | Primarily UK, with US and Australia | Training and competition trail running | Analytical cross-sectional; Questionnaire/self-report; Previous 12 months (recruited 2023). | 62 runners; 115 injuries. | Self-reported person-time | JBI-ACS; Y3/N2/U3/NA0; Include |
| S12. Ruedl et al., 2026 | Tyrol, western Austria | Primarily downhill bike-park riding | Prevalence/occurrence; Registry/administrative records; 2023–2024. | 274 injured riders; exposure denominator from one park only. | Visits/rides from one bike park | JBI-Prev; Y3/N3/U3/NA0; Include |
| S13. Westman and Björnstig, 2024 | Swedish Scandes (Norrbotten, Västerbotten, Jämtland, Dalarna) | Hiking, skiing, snowmobiling and other activities | Prevalence/occurrence; Rescue/incident records; 2018–2022. | 1,543 missions; 1,857 confirmed persons; 39 fatalities. | Case/mission count only | JBI-Prev; Y6/N1/U2/NA0; Include |
| S14. Westensee et al., 2013 | Aconcagua Provincial Park, Argentina | Aconcagua summit attempts | Prevalence/occurrence; Aconcagua permit/fatality registry; 2001–2012. | 42,731 summit attempts; 33 deaths. | Summit permits | JBI-Prev; Y6/N0/U2/NA1; Include |
| S15. Samara et al., 2024 | Palm Springs, California, USA | Rock-climbing falls from height | Case series; Hospital/trauma registry; 2016–2021. | 75 climbers. | Case/mission count only | JBI-CS; Y7/N1/U2/NA0; Include |
| S16. Gasser, 2022 | Swiss Alps | Alpine climbing/mountaineering emergencies | Analytical cross-sectional; Swiss Alpine Club mountain-accident registry; 2009–2020. | 4,687 emergencies (analytic table: 4,691); 1,951 stranded cases. | Case/mission count only | JBI-ACS; Y3/N2/U3/NA0; Include |
| S17. Castellani et al., 2018 | Styria, Austria | Skiing/snowboarding and other slope activities | Prevalence/occurrence; Ski-patrol records + exposure/control data; 2007/08 and 2014/15 seasons. | 1,029 injuries (2007/08) and 1,844 (2014/15); total 2,873. | Skier-days/lift transports | JBI-Prev; Y3/N2/U4/NA0; Include |
| S18. Pasquier et al., 2017 | Switzerland | Summer alpine climbing/hiking versus winter touring/riding | Analytical cross-sectional; National avalanche registry; 1984–2014. | 482 fatal avalanches; 21 summer vs 461 winter; 655 fatalities (40 summer). | Case/mission count only | JBI-ACS; Y3/N2/U3/NA0; Include |
| S19. Rauch et al., 2022 | Switzerland | Mainly ski touring/hiking and other avalanche activities. | Cohort; National avalanche registry; 1998–2020. | 4,270 victims; 3,892 analyzed; 957 completely buried (model n=952). | Case/mission count only | JBI-Cohort; Y9/N1/U0/NA1; Include |
| S20. Brillhart et al., 2025 | Aconcagua Provincial Park, Argentina | Aconcagua summit attempts | Prevalence/occurrence; Aconcagua permit/fatality registry; 2013–2024. | 29,397 summit attempts; 21 deaths. | Summit permits | JBI-Prev; Y4/N0/U4/NA1; Include |
| S21. Heggie & Amundson, 2009 | United States | Hiking and other national-park outdoor activities | Prevalence/occurrence; National-park SAR/registration records; 1992–2007. | 65,439 incidents/78,488 people; detailed 2005: 2,430 incidents/3,297 people. | Case/mission count only | JBI-Prev; Y5/N0/U3/NA1; Include |
| S22. Deeb et al., 2018 | Slovenia | Activity not recorded | Prevalence/occurrence; Rescue/incident records; 2011–2015. | ~413 operations/year; 2015: 434 operations, 470 people, 190 helicopter rescues. | Case/mission count only | JBI-Prev; Y7/N1/U1/NA0; Include |
| S23. Gross et al., 2021 | Canton of Bern, Switzerland | Ski/snowboard touring and freeriding | Analytical cross-sectional; Registry/administrative records; 2001–2019. | 62 identified; 59 reviewed; 51 accidental and 8 natural deaths. | Case/mission count only | JBI-ACS; Y2/N2/U4/NA0; Include |
| S24. Boshielo et al., 2025 | South Africa | Trail/ultra-trail running | Analytical cross-sectional; Participant survey + race medical records; 15 Nov 2020. | 285/331 consented (86.1%); 89 runners had 131 encounters (49 injuries, 82 illnesses). | Race participants | JBI-ACS; Y3/N3/U2/NA0; Include |
| S25. McIntosh et al., 2008 | Denali National Park, Alaska, USA | Denali climbing | Cohort; National-park SAR/registration records; 1903–2006. | 31,201 climbers; 96 deaths. | Registered climbers | JBI-Cohort; Y5/N3/U3/NA0; Include |
| S26. DeLoughery, 2022 | United States | Hiking/climbing and related mountain activities | Case series; National-park SAR/registration records; 1947–2018. | 2,799 persons; 1,204 deaths; 1,595 nonfatally injured. | Partial climber counts | JBI-CS; Y6/N2/U2/NA0; Seek further info |
| S27. Festini Capello et al., 2025 | South Tyrol, Italy | Skiing/snowboarding | Analytical cross-sectional; Questionnaire/self-report; 8 Dec 2023–31 Mar 2024. | 579 consecutive questionnaires/patients. | Case/mission count only | JBI-ACS; Y4/N0/U4/NA0; Include |
| S28. Woyke et al., 2024 | Austria | MTB accidents | Analytical cross-sectional; Austrian mountain-accident/police registry; 2006–2018. | 4,973 accidents involving 5,103 people; 5,095 analyzed. | Case/mission count only | JBI-ACS; Y3/N2/U3/NA0; Include |
| S29. Soulé et al., 2017 | France | Multi-activity mountain recreation | Prevalence/occurrence; Rescue/incident records; 1971–2015. | Police: 30,830 victims (2008–2012); NSOSM 2012: 5,389 interventions/6,362 people. | Case/mission count only | JBI-Prev; Y1/N3/U4/NA1; Include |
| S30. Hohlrieder et al., 2005 | Austrian Alps | Backcountry ski touring and off-piste skiing/snowboarding | Analytical cross-sectional; Austrian mountain-accident/police registry; 1994–2003. | 194 accidents; 278 totally buried victims. | Case/mission count only | JBI-ACS; Y6/N1/U1/NA0; Include |
| S31. Dodge et al., 2026 | Nepal/China (Everest) | Everest expeditions | Cohort; Himalayan Database + supplementary reports; 1921–2024. | 30,385 climbers above base camp; 12,881 summit ascents; 309 deaths. | Climbers/summit descents | JBI-Cohort; Y8/N1/U1/NA1; Include |
| S32. McClung, 2016 | High mountains of Asia | Expedition mountaineering | Case series; Registry/administrative records; 2014. | ~700 deaths; 688 regional deaths; 616 classified by mechanism. | Case/mission count only | JBI-CS; Y4/N3/U3/NA0; Seek further info |
| S33. Brandão et al., 2026 | International (mainly Spain/Portugal) | Canyoning | Analytical cross-sectional; Questionnaire/self-report; Events ≤15 years before 2022 survey. | 179 participants/events; 163 injuries classified. | Case/mission count only | JBI-ACS; Y3/N2/U3/NA0; Include |
| S34. Ciesa et al., 2015 | Veneto Prealps, Italy | Predominantly hiking and mountaineering | Prevalence/occurrence; Rescue/incident records; 1992–2012. | 835 operations; 942 rescued/assisted people. | Case/mission count only | JBI-Prev; Y5/N2/U2/NA0; Include |
| S35. Mort and Godden, 2010 | United Kingdom | Hillwalking, climbing, mountain biking and other mountain activities | Prevalence/occurrence; Rescue/incident records; 2002–2006. | 6,814 incidents/7,995 people; 3,152 detailed reports analyzed. | Case/mission count only | JBI-Prev; Y2/N2/U5/NA0; Include |
| S36. Faulhaber et al., 2017 | Austrian Alps | Mountain hiking falls | Analytical cross-sectional; Austrian mountain-accident/police registry; 2006–2014. | 5,368 accidents; 5,665 victims after 312/5,680 events excluded. | Case/mission count only | JBI-ACS; Y3/N2/U3/NA0; Include |
| S37. Chen et al., 2020 | Chongli, China | Alpine skiing and snowboarding | Analytical cross-sectional; Resort clinic + skier-day data; 1 Nov 2018–7 Apr 2019. | 753 injuries; 388,606 visits and 379,503 skier days. | Skier-days | JBI-ACS; Y4/N2/U2/NA0; Include |
| S38. Madsen et al., 2025 | United States | Rock climbing/bouldering (indoor/outdoor) | Analytical cross-sectional; NEISS emergency-department surveillance; 2013–2022. | 1,372 verified cases from 1,397 query hits; weighted national estimate 49,737. | Weighted case estimate; no activity exposure | JBI-ACS; Y5/N2/U1/NA0; Include |
| S39. Ströhle et al., 2020 | Austria | Protected-route climbing | Analytical cross-sectional; Police incident records; 2008–2018. | 1,231 emergencies involving 1,684 persons. | Case/mission count only | JBI-ACS; Y3/N2/U3/NA0; Include |
| S40. Faulhaber et al., 2020 | Tyrol, Austria | Nonfatal mountain-hiking falls | Case series; Austrian mountain-accident/police registry; 2016–2018. | 405 respondents; approximately 50% response rate. | Case/mission count only | JBI-CS; Y7/N2/U1/NA0; Include |
| S41. Pocecco et al., 2022 | Austrian Alps | Recreational MTB | Prevalence/occurrence; Austrian mountain-accident/police registry; 2006–2021. | 7,675 accidents, 7,892 riders; 97 fatal deaths. | Case/mission count only | JBI-Prev; Y6/N0/U3/NA0; Include |
| S42. Gasser, 2022 | Swiss Alps | Alpine routes/glacier mountaineering | Prevalence/occurrence; Swiss Alpine Club mountain-accident registry; 2009–2021. | 5,020 emergencies; 303 fatal events. | Case/mission count only | JBI-Prev; Y5/N0/U3/NA1; Include |
| S43. Koehne et al., 2025 | United States | Mountain/all-terrain bicycle injury | Analytical cross-sectional; NEISS emergency-department surveillance; 2013–2022. | 2,543 cases; weighted national estimate 109,558. | Weighted case estimate; no activity exposure | JBI-ACS; Y5/N2/U1/NA0; Include |
| S44. Lin et al., 2026 | United States | Rock climbing excluding mountaineering/ice climbing | Analytical cross-sectional; NEISS emergency-department surveillance; 2014–2023. | 1,306 raw cases; estimated 47,251 injuries. | US population; no activity exposure | JBI-ACS; Y5/N2/U1/NA0; Include |
| S45. Chu Kwan et al., 2024 | British Columbia, Canada | Off-road MTB causing acute SCI | Case series; Hospital/trauma registry; 2008–2022. | 58 patients. | Case/mission count only | JBI-CS; Y8/N0/U2/NA0; Include |
| S46. Niemann et al., 2022 | United States | Backcountry/sidecountry ski-snowboard and snowmobile/snowbike | Case series; Registry/administrative records; 2009/10–2018/19 seasons. | 205 fatalities: 32 sidecountry, 91 ski/snowboard, 82 snowmobile/snowbike; 53 excluded. | Case/mission count only | JBI-CS; Y4/N3/U3/NA0; Seek further info |
| S47. Owen et al., 2024 | United States | Hiking, trail running and mountain biking | Analytical cross-sectional; NEISS emergency-department surveillance; 2002–2021. | 9,835 raw injuries; national estimate 1,146,630. | Weighted case estimate; no activity exposure | JBI-ACS; Y4/N2/U2/NA0; Include |
| S48. Rausch et al., 2024 | Austrian Alps | Mountain hiking | Analytical cross-sectional; Austrian mountain-accident/police registry; 2015–2021. | 7,368 accidents/7,552 victims analyzed; 624 accidents/700 victims excluded. | Case/mission count only | JBI-ACS; Y4/N2/U2/NA0; Include |
| S49. Rauch et al., 2024 | Switzerland | Critical burial among backcountry/out-of-bounds skiers | Cohort; National avalanche registry; 1981–2020. | 1,643 critically buried; 878 survivors and 765 nonsurvivors. | Case/mission count only | JBI-Cohort; Y9/N0/U2/NA0; Include |
| S50. Ruedl et al., 2026 | Western Tyrol, Austria | Electric versus traditional MTB | Analytical cross-sectional; Rescue/incident records; 2023–2024. | 316 injured riders; 35 e-MTB (11.1%). | Case/mission count only | JBI-ACS; Y4/N1/U3/NA0; Include |
| S51. Soulé et al., 2017 | France | Multi-activity mountain recreation | Prevalence/occurrence; Six shortlisted sources from >20 datasets plus 46 stakeholder interviews/feedback seminar.; 1971–2013. | Multiple sources; largest police dataset 30,830 victims; 46 stakeholder interviews. | Case/mission count only | JBI-Prev; Y1/N4/U3/NA1; Include |
| S52. Vanpoulle et al., 2021 | French Alps, France | Mountaineering | Analytical cross-sectional; French national police rescue registry; 2008–2018. | 7,819 evacuated persons; 3,641 impacted (46.6%); 377 deaths (4.8%, traumatic and nontraumatic combined). | Case/mission count only | JBI-ACS; Y3/N2/U3/NA0; Include |
| S53. Chen et al., 2025 | Taiwan | Predominantly hiking/trekking | Cohort; Rescue/incident records; 2017–2022. | 783 MROs, 965 rescued victims; 1,427,403 registered trekkers. | Registered trekkers | JBI-Cohort; Y7/N2/U2/NA0; Include |
| S54. Pietsch et al., 2019 | Zermatt region, Switzerland | HEC hoist/longline rescue | Case series; Rescue/incident records; Jan 2010–Sep 2016. | 842/1,137 HEC protocols (74%). | Case/mission count only | JBI-CS; Y6/N3/U1/NA0; Include |
| S55. Sato & Fukazawa, 2025 | Nagano Prefecture, Japan | Mountain climbing/hiking across Nagano mountain ranges. | Prediction model; Police incident records; 2014–2023. | 2,569–2,596 cases reported inconsistently in the source. | Case/mission count only | PROBAST; high RoB; high applicability concern |
| S56. Breulmann et al., 2024 | Schladming, Austria | Recreational downhill mountain biking | Prevalence/occurrence; Hospital records + lift exposure data; 2020–2021. | 936 injured riders; 89,658 guests/407,074 rides (season totals); demographic table n=650. | Lift guests/rides | JBI-Prev; Y3/N4/U2/NA0; Seek further info |
| S57. Sulheim et al., 2011 | Norway | Alpine skiing, telemark skiing, snowboarding | Case-control; Ski-patrol records + exposure/control data; 2002. | 3,277 injury cases and 2,992 controls. | Case-control comparator | JBI-CC; Y9/N0/U1/NA0; Include |
| S58. Neumair et al., 2022 | Austria | Summer and winter mountain activities | Ecological analytical cross-sectional/time-series study; Registry/administrative records; 2006–2018. | 3,285 fatal accidents (1,817 summer; 1,468 winter) retained from 3,466; daily weather at 43 stations over 12 years. | Weather station-days; no activity exposure | JBI-ACS; Y5/N0/U3/NA0; Include |
| S59. Wild, 2008 | Canada (Alberta/British Columbia) | Multi-activity mountain recreation | Prevalence/occurrence study (rescue case series); National-park SAR/registration records; 2003–2006. | 317 rescue operations involving 406 persons; 366 survivors and 40 fatalities. | Case/mission count only | JBI-Prev; Y5/N1/U3/NA0; Include |
| S60. Wagner et al., 2023 | Tyrol, Austria | Alpine skiing and snowboarding (not differentiated in results). | Prevalence/occurrence; State emergency dispatch center plus electronic skier-day tracking from the chamber of commerce/ski resorts.; Nov 2017–Apr 2022. | 43,283 patients; 98.1 million skier-days; detailed clinical subset ~9,103. | Skier-days | JBI-Prev; Y5/N2/U2/NA0; Include |
| S61. Tomasi et al., 2026 | Italy | Canyoning rescue | Analytical cross-sectional/registry study; Rescue/incident records; 2009–2023. | 344 rescue operations involving 569 patients. | Case/mission count only | JBI-ACS; Y4/N1/U3/NA0; Include |
| S62. Mueller et al., 2019 | Alps (participants from 8 countries) | Ski touring | Cohort; Questionnaire/self-report; Nov 2015–May 2016. | 191 participants; 3,900 tours; 10,955 exposure-hours; 27 injury events. | Participants/tours/exposure-hours | JBI-Cohort; Y6/N2/U3/NA0; Include |
| S63. Ruedl et al., 2014 | Austria | Alpine skiing and snowboarding | Prevalence/occurrence; Questionnaire/self-report; Winter 2012/13. | 7,325 injured participants; ~15 million skier-days; complete subsample n=1,451. | Skier-days | JBI-Prev; Y4/N1/U4/NA0; Include |
| S64. Migiel et al., 2026 | Polish Tatra Mountains | Mostly hiking; also skiing, cycling, climbing, caving and others | Analytical cross-sectional; TOPR electronic rescue registry; 2015–2024. | 8,908 missions; 9,695 victims recorded; 9,477 individuals analyzed. | Park visitors | JBI-ACS; Y4/N2/U2/NA0; Include |
| S65. Gasser, 2019 | Swiss Alps | Mountain hiking | Analytical cross-sectional; Swiss Alpine Club mountain-accident registry; 2009–2018. | 11,220 rescued/recovered; 10,185 analyzed. | Case/mission count only | JBI-ACS; Y4/N3/U1/NA0; Include |
| S66. Durand-Bechu et al., 2014 | France | Roped climbing and bouldering, indoors and outdoors | Prevalence/occurrence; Federation accident declarations; 2004–2011. | 1,128 accidents; federation membership 57,648–72,190. | Federation members/estimated hours | JBI-Prev; Y4/N1/U4/NA0; Include |

**Abbreviations:** JBI-ACS, JBI analytical cross-sectional tool; JBI-Prev, JBI prevalence tool; JBI-CS, JBI case-series tool; JBI-Cohort, JBI cohort tool; JBI-CC, JBI case-control tool; PROBAST, Prediction model Risk Of Bias ASsessment Tool; Y, yes; N, no; U, unclear; NA, not applicable; RoB, risk of bias; NR, not reported.

Note: Y/N/U/NA counts are descriptive item-response profiles, not quality scores or grades, and are not numerically comparable across instruments. JBI Include/Seek further info entries are tool-specific review decisions rather than high/low quality ratings; all eligible studies remained included. PROBAST conclusions retain its official risk-of-bias and applicability terminology. Participation exposure refers to an activity-based denominator (people, time, visits, rides, permits, or equivalent); case or mission counts alone are not participation exposure.

**Supplementary Table S2. Database-specific search strategies**

| **Source** | **Fields** | **Search expression** |
| --- | --- | --- |
| PubMed (n=896) | Title/Abstract | ((mountain OR mountains OR alpine OR wilderness OR backcountry OR "high altitude" OR "high-altitude" OR trail OR canyon) AND (hiking OR mountaineering OR climbing OR "rock climbing" OR "via ferrata" OR skiing OR "mountain biking" OR "trail running" OR canyoning OR snowmobiling OR snowmobile OR avalanche) AND (accident OR accidents OR injury OR injuries OR trauma OR illness OR fatality OR fatalities OR mortality OR death OR rescue OR "search and rescue" OR evacuation OR survival OR prevention)) |
| Web of Science Core Collection (n=1,772) | Topic (TS) | TS=((mountain* OR alpine OR wilderness OR backcountry OR "high altitude" OR trail OR canyon*) AND (hiking OR mountaineering OR climbing OR "rock climbing" OR "via ferrata" OR skiing OR "mountain biking" OR "trail running" OR canyoning OR snowmobil* OR avalanche*) AND (accident* OR injur* OR trauma OR illness OR fatalit* OR mortality OR death OR rescue OR "search and rescue" OR evacuation OR survival OR prevention)) |
| Scopus (n=2,157) | TITLE-ABS-KEY | TITLE-ABS-KEY((mountain* OR alpine OR wilderness OR backcountry OR "high altitude" OR trail OR canyon*) AND (hiking OR mountaineering OR climbing OR "rock climbing" OR "via ferrata" OR skiing OR "mountain biking" OR "trail running" OR canyoning OR snowmobil* OR avalanche*) AND (accident* OR injur* OR trauma OR illness OR fatalit* OR mortality OR death OR rescue OR "search and rescue" OR evacuation OR survival OR prevention)) |
| EBSCOhost, including SPORTDiscus (n=317) | Title/Abstract | ((TI(mountain* OR alpine OR wilderness OR backcountry OR "high altitude" OR trail OR canyon*) OR AB(mountain* OR alpine OR wilderness OR backcountry OR "high altitude" OR trail OR canyon*)) AND (TI(hiking OR mountaineering OR climbing OR "rock climbing" OR "via ferrata" OR skiing OR "mountain biking" OR "trail running" OR canyoning OR snowmobil* OR avalanche*) OR AB(hiking OR mountaineering OR climbing OR "rock climbing" OR "via ferrata" OR skiing OR "mountain biking" OR "trail running" OR canyoning OR snowmobil* OR avalanche*)) AND (TI(accident* OR injur* OR trauma OR illness OR fatalit* OR mortality OR death OR rescue OR "search and rescue" OR evacuation OR survival OR prevention) OR AB(accident* OR injur* OR trauma OR illness OR fatalit* OR mortality OR death OR rescue OR "search and rescue" OR evacuation OR survival OR prevention))) |
